# Burosumab use in fibroblast growth factor-23-mediated hypophosphatemia in McCune-Albright syndrome/fibrous dysplasia

**DOI:** 10.1210/jcemcr/luag086

**Published:** 2026-05-06

**Authors:** Marah Alsayed Hasan, Mona Al Mukaddam

**Affiliations:** Division of Endocrinology, Diabetes and Metabolism, Hospital of the University of Pennsylvania, Philadelphia, PA 19104, USA; Division of Endocrinology, Diabetes and Metabolism, Hospital of the University of Pennsylvania, Philadelphia, PA 19104, USA

**Keywords:** burosumab, FGF-23, hypophosphatemia, fibrous dysplasia, bone pain

## Abstract

McCune-Albright syndrome (MAS) is a rare disorder characterized by fibrous dysplasia (FD), café-au-lait spots, and hyperfunctioning endocrinopathies. FD lesions can overproduce fibroblast growth factor-23 (FGF-23), leading to renal phosphate wasting, hypophosphatemia, and impaired bone mineralization. Conventional treatment with oral phosphate and calcitriol is limited by gastrointestinal intolerance. Burosumab, a monoclonal antibody against FGF-23, is approved for X-linked hypophosphatemia and tumor-induced osteomalacia and has shown promise in case reports of pediatric and adult patients with MAS-related FGF-23-mediated hypophosphatemia. We describe a 46-year-old woman with MAS and extensive FD who presented with worsening bone pain and FGF-23-mediated hypophosphatemia. She was started on phosphate and calcitriol with improvement in pain. Due to failure to achieve treatment targets, she was transitioned to burosumab 0.50 mg/kg injections every 4 weeks with normalization of serum phosphorus but persistent elevation of alkaline phosphatase. This case represents the second reported adult and oldest patient treated with burosumab for MAS-related FGF-23-mediated hypophosphatemia after failure of conventional therapy. Although its use in MAS remains off-label, burosumab may provide a more targeted and better-tolerated therapeutic option.

## Introduction

McCune-Albright syndrome (MAS) is a rare disorder characterized by fibrous dysplasia (FD), hyperfunctioning endocrinopathies, and café-au-lait macules [1-3]. It results from a postzygotic activating pathogenic variant in *GNAS*, which encodes the stimulatory G-protein α-subunit, leading to constitutive activation of cyclic adenosine monophosphate signaling [1-3]. This mechanism drives hormone overproduction, skin hyperpigmentation, and abnormal differentiation of skeletal progenitor cells within the affected bone [2]. MAS is diagnosed clinically in individuals with extra-skeletal manifestations, with or without FD [1-3].

FD lesions arise early in life and consist of abnormally differentiated bone stromal cells that form structurally disorganized and poorly mineralized bone, predisposing individuals to skeletal deformities, fractures, and chronic pain [1-3]. FD lesions overproduce fibroblast growth factor-23 (FGF-23), leading to renal phosphate wasting in half of the individuals with FD/MAS [1-4]. The degree of FGF-23 overproduction and the resulting phosphate wasting correlates with skeletal disease burden, with frank hypophosphatemia occurring with extensive skeletal involvement [1-4]. Conventional treatment of FGF-23-mediated hypophosphatemia includes oral phosphate and active vitamin D analogs [1].

Burosumab, an IgG-1 monoclonal antibody against FGF-23, is approved for the treatment of X-linked hypophosphatemia (XLH) and tumor-induced osteomalacia (TIO) and has shown promise in pediatric cases and one adult with MAS [5-12]. A recent National Institutes of Health (NIH) Phase 2 trial evaluated the safety and efficacy of burosumab in restoring bone turnover and maintaining high-normal serum phosphorus levels in 12 patients aged ≥1 year with FD and FGF-23-mediated hypophosphatemia, although the results remain unpublished [13]. Given the rarity of the disease and the paucity of adult treatment data, we report on our clinical experience of the oldest adult patient reported to date with MAS/FD and FGF-23-mediated hypophosphatemia treated with burosumab. Treatment normalized serum phosphorus and improved clinical outcomes.

## Case presentation

A 46-year-old woman with MAS presented to our endocrinology clinic for evaluation of her extensive FD-related bone pain. She was diagnosed with precocious puberty at 7 months of age after presenting with vaginal bleeding, and a right ovarian mass was surgically removed. MAS was confirmed at age 2 based on café-au-lait macules and widespread skeletal FD. She sustained recurrent childhood fractures requiring orthopedic surgeries and became wheelchair-bound by age 16 due to impaired mobility and frequent falls. She developed hyperthyroidism in her twenties, which was managed with methimazole. She reported kidney stones in her twenties that resolved without intervention. At the time of her presentation, she was under evaluation by oral and maxillofacial surgery for mandibular FD requiring debridement and excision. Functionally, she reported moderate difficulty climbing stairs and driving long distances with pedal extenders due to pain and weakness, and she required assistance lifting heavy objects.

## Diagnostic assessment

Her physical examination showed short stature (height 4 ft 9 in), marked mandibular enlargement, bowing of the lower extremities, and café-au-lait spots on the back that crossed the midline. She was wheelchair-bound.

**Figure 1:**
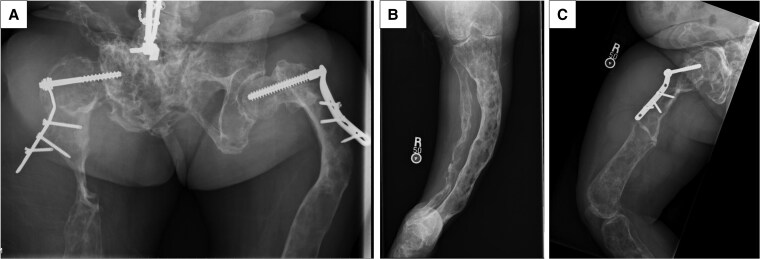
Plain radiographs of (A) the hips, (B) the right tibia and fibula, and (C) the right femur in a patient with McCune-Albright syndrome and polyostotic fibrous dysplasia. The images demonstrate extensive mosaic, ground-glass, and lytic changes involving all the visualized osseous structures, consistent with fibrous dysplasia. There is an increased risk of pathologic fracture, especially of the right femur and tibia-fibula noting cortical destruction with minimal residual bone mass. Bilateral proximal femoral fixation hardware is present with radiographic features suspicious for hardware loosening.

Initial laboratory evaluation demonstrated low-normal phosphorus, low insulin-like growth factor 1, and markedly elevated bone turnover markers, including C-telopeptide (CTX) and alkaline phosphatase (ALP) (Table 1). Thyroid function, prolactin, and 25-hydroxyvitamin D levels were within normal limits, and screening for Cushing syndrome was negative. Renal phosphate wasting was assessed using the tubular maximum reabsorption of phosphate to glomerular filtration rate (TmP/GFR) and FGF-23 levels. The low TmP/GFR with elevated FGF-23 confirmed renal phosphate wasting (Table 1). Urine studies showed hypercalciuria, hypercitraturia, and elevated calcium phosphate saturation (Table 2). Skeletal radiographs demonstrated extensive FD with cortical thinning and hardware loosening (Fig. 1).

**Table 1:** Laboratory evaluation at initial presentation and after 12 months of burosumab therapy in a patient with MAS/FD

Parameter	Reference range	October 2023	December 2025
Serum calcium	8.60-10.20 mg/dL (2.20-2.60 mmol/L)	9.40 mg/dL (2.35 mmol/L)	9.30 mg/dL (2.32 mmol/L)
Serum albumin	3.60-5.10 g/dL (35-50 g/L)	4.30 g/dL (43 g/L)	4.50 g/dL (45 g/L)
Serum phosphorus	Quest assay: 2.50-4.50 mg/dL (0.81-1.45 mmol/L) LabCorp assay: 3.00-4.50 mg/dL (0.97-1.45 mmol/L)	2.40 mg/dL (0.77 mmol/L)*^a^*	3.40 mg/dL (1.10 mmol/L)*^b^*
Serum creatinine	0.50-0.99 mg/dL (44-97 µmol/L)	0.25 mg/dL (22 µmol/L)	0.31 mg/dL (27 µmol/L)
25-(OH)D	30-100 ng/mL (75-250 nmol/L)	30 ng/mL (75 nmol/L)	35.3 ng/mL (88 nmol/L)
1,25-(OH)_2_D	18-72 pg/mL (43-143 pmol/L)	37 pg/mL (89 pmol/L)	ND
iPTH	16-77 pg/mL (1.7-8.2 pmol/L)	65 pg/mL (6.9 pmol/L)	ND
ALP	31-125 U/L (0.5-2.1 µkat/L)	455 U/L (7.6 µkat/L)	343 U/L (5.7 µkat/L)
Serum CTX	50-465 pg/mL (50-465 ng/L)	1250 pg/mL (1250 ng/L)	ND
FGF-23	<180 RU/mL	229 RU/mL	
TmP/GFR	2.60-3.80 mg/dL (0.84-1.23 mmol/L)	2.17 mg/dL (0.70 mmol/L)	

Values in parentheses are International System of Units (SI).

Abbreviations: 1,25-(OH)_2_D, 1,25-dihydroxyvitamin D; 25-(OH)D, 25-hydroxyvitamin D; ALP, alkaline phosphatase; CTX, C-terminal telopeptide of type I collagen; FGF-23, fibroblast growth factor-23; iPTH, intact parathyroid hormone; ND, not done; TmP/GFR, tubular maximum reabsorption of phosphate to glomerular filtration rate.

^*a*^Quest assay.

^*b*^LabCorp assay.

**Table 2:** Litholink 24-hour urine analysis at initial presentation in a patient with MAS/FD

	Reference range	November 2023
Urine volume	500-4000 mL/24 hours (0.50-4.00 L/day)	1240 mL/24 hours (1.24 L/day)
Urine pH	5.80-6.20	6.50
Calcium	<200 mg/24 hours (<5.0 mmol/24 hours)	296 mg/24 hours (7.38 mmol/24 hours)
Citrate	>550 mg/24 hours (>2.86 mmol/24 hours)	638 mg/24 hours (3.33 mmol/24 hours)
Oxalate	20-40 mg/24 hours (0.22-0.44 mmol/24 hours)	12 mg/24 hours (0.14 mmol/24 hours)
Phosphorus	600-1200 mg/24 hours (19.37-38.74 mmol/24 hours)	554 mg/24 hours (17.89 mmol/24 hours)
Uric acid	<750 mg/24 hours (<4.46 mmol/24 hours)	502 mg/24 hours (2.99 mmol/24 hours)
Sodium	50-150 mmol/24 hours	119 mmol/24 hours
Calcium oxalate saturation	6-10	5.34
Calcium phosphate saturation	0.50-2.00	3.46
Creatinine/kg body weight	8.70-20.30 mg/24 hours/kg (0.08-0.18 mmol/24 hours/kg)	11.70 mg/24 hours/kg (0.10 mmol/24 hours/kg)
Calcium/creatinine ratio	51-263 mg/g creatinine	700 mg/g creatinine

Values in parentheses are International System of Units (SI).

## Treatment

She was initiated on oral calcium carbonate 600 mg daily, calcitriol 0.25 mcg daily, and potassium phosphate-sodium phosphate 155-852-130 mg (250 mg elemental phosphate) twice daily, with plans to consider intravenous zoledronic acid for her bone pain. However, bisphosphonates and denosumab were deferred due to concerns regarding postoperative healing after planned mandibular surgery, hypophosphatemia, and rebound hypercalcemia and fracture risk. She reported meaningful improvement in bone pain after starting phosphate and calcitriol. Despite escalation to high-dose therapy (calcitriol up to 3 mcg daily and phosphate up to 4000 mg daily), her serum phosphorus remained below the treatment target, ranging from 2.30 to 2.70 mg/dL (SI: 0.74-0.87 mmol/L) (reference range: 3.00-4.50 mg/dL [SI: 0.97-1.45 mmol/L]) (Table 3). Dose escalation was limited by recurrent diarrhea, precluding the achievement of target serum phosphorus levels within or near the lower limit of the normal range.

**Table 3:** Longitudinal laboratory measures and medication regimen in the treatment of a patient with MAS/FD

Date	Serum phosphorus (Quest) 2.50-4.50 mg/dL (0.81-1.45 mmol/L)	Serum phosphorus (LabCorp) 3.00-4.50 mg/dL (0.97-1.45 mmol/L)	Alkaline phosphatase 31-125 U/L (0.52-2.08 µkat/L)	Medication regimen
November 14, 2023	2.40 mg/dL (0.77 mmol/L)	—	—	Calcium carbonate 600 mg once daily, calcitriol 0.25 mcg once daily, potassium phosphate–sodium phosphate 250 mg elemental twice daily
December 28, 2023	2.90 mg/dL (0.94 mmol/L)	—	473 U/L (7.9 µkat/L)	—
February 28, 2024	2.30 mg/dL (0.74 mmol/L)	—	473 U/L (7.9 µkat/L)	Stopped medications due to hives; restarted calcium carbonate 600 mg once daily, calcitriol 0.25 mcg twice daily, potassium phosphate–sodium phosphate 250 mg elemental 3 times daily
March 12, 2024	2.60 mg/dL (0.84 mmol/L)	—	397 U/L (6.6 µkat/L)	—
June 6, 2024	—	2.40 mg/dL (0.77 mmol/L)	—	Not taking medications regularly due to gastrointestinal side effects; calcium carbonate 600 mg once daily, calcitriol 0.25 mcg twice daily, potassium phosphate–sodium phosphate 250 mg elemental 3 times daily every other day
August 5, 2024	—	2.30 mg/dL (0.74 mmol/L)	—	—
September 12, 2024	—	2.70 mg/dL (0.87 mmol/L)	—	Calcium carbonate 600 mg once daily, calcitriol 1 mcg 3 times daily, potassium phosphate–sodium phosphate 500 mg elemental 3 times daily
October 29, 2024	—	2.50 mg/dL (0.81 mmol/L)	372 U/L (6.2 µkat/L)	—
November 26, 2024	—	2.40 mg/dL (0.77 mmol/L)	—	Not adherent due to gastrointestinal side effects; calcium carbonate stopped; calcitriol 2 mcg once daily, potassium phosphate–sodium phosphate 500 mg elemental (2 tablets) 4 times daily
December 1, 2024	—	—	—	Phosphate and calcitriol stopped 2 weeks prior to burosumab initiation. burosumab 20 mg (0.50 mg/kg) subcutaneously every 4 weeks
January 29, 2025	—	3.50 mg/dL (1.13 mmol/L)	466 U/L (7.7 µkat/L)	Burosumab continued at same dose
February 27, 2025	—	3.50 mg/dL (1.13 mmol/L)	449 U/L (7.5 µkat/L)	Burosumab continued at same dose
March 27, 2025	—	3.40 mg/dL (1.10 mmol/L)	456 U/L (7.6 µkat/L)	Burosumab continued at same dose
September 20, 2025	—	2.40 mg/dL (0.77 mmol/L)	304 U/L (5.1 µkat/L)	Burosumab continued at same dose
December 4, 2025	—	3.70 mg/dL (1.19 mmol/L)	364 U/L (6.1 µkat/L)	Burosumab increased to 30 mg (1 mg/kg) subcutaneously every 4 weeks

Values in parentheses are International System of Units (SI).

After a 2-week washout of conventional therapy, burosumab 0.50 mg/kg (20 mg) was initiated subcutaneously every 4 weeks. Her serum phosphorus normalized after 2 doses, but her ALP remained elevated at 343 U/L (SI: 5.7 µkat/L) (reference range: 31-125 U/L [SI: 0.5-2.1 µkat/L]) despite 12 months of burosumab.

## Outcome and follow-up

She continued burosumab 20 mg every 4 weeks for 12 months with rapid normalization of her serum phosphorus but without a significant reduction in her ALP. Serum calcium, phosphorus, and ALP were monitored monthly initially and less frequently after stabilization of lab values (Table 3). She reported marked improvement in her quality of life, including reduced pill burden and resolution of diarrhea off phosphate therapy. She also described substantial improvement in pain by 3 months and progressive gains in lower extremity strength by 6-9 months, resulting in greater independence in daily activities. Zoledronic acid is no longer being considered for pain management.

After 2 burosumab doses, she presented to the emergency department with flank pain and was found to have a 7-mm nonobstructive right renal stone that self-resolved. This was attributed to pre-existing hypercalciuria on high-dose conventional therapy rather than an adverse effect of burosumab. During this evaluation, a left hepatic mass was incidentally identified. Parasitic testing was negative, and subsequent surgical resection confirmed a benign cyst, with ALP improving to 304 U/L (SI: 5.1 µkat/L).

Her burosumab dose was increased to 30 mg (1 mg/kg) every 4 weeks after 12 months to target a high-normal serum phosphorus concentration.

## Discussion

FD, a manifestation of MAS, is a skeletal disease characterized by replacement of normal bone with fibro-osseous tissue composed of immature osteogenic cells and disorganized trabeculae [4]. The mechanism of hypophosphatemia in FD was elucidated when Collins et al identified renal phosphate wasting in approximately half of the patients with FD/MAS, correlating with skeletal disease burden and bone turnover markers [3]. Subsequent work by Riminucci et al demonstrated overexpression of FGF-23 within FD lesions, resulting in hypophosphatemia and predisposing the poorly mineralized bone to increased skeletal morbidity, including fractures, scoliosis, and orthopedic surgery [14].

Conventional treatment with oral phosphate and active vitamin D can improve serum phosphorus but is often limited by gastrointestinal intolerance, high pill burden, nephrocalcinosis, hypercalciuria, and hyperparathyroidism [15]. Burosumab, a monoclonal antibody against FGF-23, is approved in XLH and TIO, where clinical trials have demonstrated normalization of serum phosphorus and improvements in fracture healing, pain, stiffness, and physical function, with a favorable safety profile [5, 16-19]. The FGF-23-mediated hypophosphatemia in XLH and TIO shares a similar pathophysiology with MAS/FD, providing a strong rationale for evaluating burosumab in patients with MAS/FD.

Gladding et al reported the first case of burosumab use in a 7-year-old boy with FD/MAS and refractory hypophosphatemia [7]. Subsequent case reports have supported the efficacy of burosumab in MAS-related hypophosphatemia (Table 4) [8-12]. Across published cases, burosumab consistently normalized serum phosphorus, reduced or normalized ALP, and improved bone pain with minimal adverse effects (Table 4) [7-12].

**Table 4:** Published case reports of pediatric and adult patients with FD/MAS and FGF-23-mediated hypophosphatemia treated with burosumab

Case	Patient age	Baseline phosphorus	Baseline ALP	Burosumab dose	Duration of therapy	Outcomes
Gladding et al [7]	7 years, 2 months	3.10 mg/dL (1.00 mmol/L) *(normal age-related range* 3.60-5.80 mg/dL (1.16-1.87 mmol/L))	800-900 U/L (13.3-15.0 µkat/L) *(normal age range* 142-335 U/L (2.5-5.6 µkat/L))	1.10 mg/kg every 2 weeks (20 mg)	17 months	Normalization of serum phosphorus; ALP improvement; improved bone pain, strength, and stamina; no fractures
Apperley and Senniappan [9]	5 years	2.97 mg/dL (0.96 mmol/L) *(normal* 2.50-4.99 mg/dL (0.81-1.61 mmol/L))	1172 IU/L (19.5 µkat/L) *(normal* 82-442 IU/L (1.4-7.4 µkat/L))	0.50 mg/kg every 2 weeks	5 weeks	Phosphorus increased to high normal range; ALP normalized
Sawamura et al [10]	11 years	3.00 mg/dL (0.97 mmol/L) *(normal* 3.60-5.50 mg/dL (1.16-1.78 mmol/L))	1761IU/L (29.4 µkat/L) *(normal* 54-315 IU/L (0.9-5.3 µkat/L))	0.80 mg/kg every 2 weeks (30 mg)	12 months	Normalization of serum phosphorus; ALP improvement; improved radiographic features of FD lesions
Barbato et al [8]	11 years	3.10 mg/dL (1.00 mmol/L) *(normal age-related range* 3.70-5.60 mg/dL (1.19-1.81 mmol/L))	2118 IU/L (35.3 µkat/L) *(normal age range* 105-280 IU/L (1.8-4.7 µkat/L))	0.80 mg/kg every 2 weeks (20 mg)	20 months*^a^*	Normalization of serum phosphorus; 50% ALP reduction (remained above normal); progression of FD lesions
Stelmachowska- Banaś et al [11]	27 years	1.18 mg/dL (0.38 mmol/L) *(normal range* 2.51-4.49 mg/dL (0.81-1.45 mmol/L))	1182 IU/L (19.7 µkat/L) *(normal range* 40-129 IU/L (0.7-2.2 µkat/L))	1 mg/kg every 4 weeks	24 months	Phosphorus normalized; significant ALP reduction; mild bone mineral density increase; reduced bone pain, improved muscle strength; no new fractures
Sakka et al [12]	10 years	2.40 mg/dL (0.77 mmol/L) *(normal range* 4-6 mg/dL (1.29-1.94 mmol/L))	736 IU/L (12.3 µkat/L) *(normal range* 60-240 IU/L (1.0-4.0 µkat/L))	0.66 mg/kg every 2 weeks (20 mg)	11 months	Phosphorus normalized; ALP normalized

Values in parentheses are International System of Units (SI). Units are reported as presented in the original publications.

Abbreviations: ALP, alkaline phosphatase; FD, fibrous dysplasia.

^*a*^Treatment interrupted for 9 months for surgery.

We report the second and oldest adult with MAS/FD and FGF-23-mediated hypophosphatemia treated with burosumab. Treatment was associated with marked improvement in quality of life, including reduced pill burden, resolution of gastrointestinal symptoms, and enhanced functional capacity, pain control, and lower extremity strength. Serum phosphorus stabilized in the low-normal range over 12 months, consistent with prior case reports (Table 3). However, ALP did not significantly decline, diverging from prior cases and possibly reflecting short treatment duration or suboptimal phosphorus targets. Gun et al described a negative correlation between serum phosphorus and skeletal morbidity in FD/MAS [14]. Patients with frank hypophosphatemia (Z-score ≤ −2) or low-normophosphatemia (Z-score > −2 to ≤ −1) exhibited higher ALP levels and greater risk of fractures, scoliosis, and orthopedic surgical intervention compared to those with high-normophosphatemia (Z-score > −1 to ≤ 2) [14]. Hypophosphatemia superimposed on dysplastic, poorly mineralized bone may therefore confer disproportionate skeletal morbidity relative to isolated FGF-23-mediated disorders [14]. While higher serum phosphorus targets may mitigate risk, optimal therapeutic targets remain undefined. Results from the NIH Phase 2 trial will be critical in guiding treatment goals, dosing, and long-term safety.

Persistently elevated ALP likely reflects high FD-related bone turnover compounded by hypophosphatemia-induced osteomalacia in the dysplastic bone [13, 20]. These overlapping processes make it challenging to determine the relative contribution of each to her ALP elevation. The decline in ALP following the hepatic cyst resection suggests a hepatic contribution to the initial lack of improvement in ALP on burosumab.

This report has several important limitations. First, parathyroid hormone (PTH) levels were not systematically monitored during burosumab therapy, limiting the assessment of potential disruptions in calcium–phosphate–PTH homeostasis during treatment. Second, the objective differentiation of skeletal vs hepatic contributions to the persistently elevated ALP was limited by the absence of a bone-specific ALP measurement. Finally, improvements in pain, functional capacity, and lower-extremity strength were assessed using patient-reported outcomes rather than standardized instruments. The lack of quantitative functional measures introduces potential reporting bias and limits objective comparison with outcomes reported in clinical trials.

This case represents the second documented adult with FD/MAS and FGF-23-mediated hypophosphatemia treated with burosumab and the oldest patient reported to date. Burosumab was well tolerated, normalized serum phosphorus, and improved functional outcomes, but did not normalize ALP after 12 months. These findings support burosumab as a feasible, though costly, option for patients with FD/MAS who are unresponsive or intolerant to conventional therapy. Burosumab is currently used off-label in this population and requires close biochemical monitoring. Further clarification of optimal phosphate targets, ALP response, FD lesion activity, and fracture risk will be essential in optimizing management. Results from the completed NIH Phase 2 trial may provide critical data to guide clinical practice and establish therapeutic parameters [13].

## Learning points

- MAS with extensive skeletal involvement may be complicated by FGF-23-mediated hypophosphatemia.
- Off-label burosumab use may improve hypophosphatemia and clinical outcomes in MAS/FD refractory to conventional therapy. However, long-term safety and efficacy remain unknown.
- ALP may remain elevated despite phosphorus normalization, reflecting complex bone metabolic dynamics in FD.

## Contributors

All authors made individual contributions to authorship. M.A.H. and M.A. were involved in the management of the patient and manuscript submission. All authors reviewed and approved the final draft.

## Data Availability

Original data generated and analyzed during this study are included in this published article.
